# DWI of Prostate Cancer: Optimal *b*-Value in Clinical Practice

**DOI:** 10.1155/2014/868269

**Published:** 2014-02-18

**Authors:** Guglielmo Manenti, Marco Nezzo, Fabrizio Chegai, Erald Vasili, Elena Bonanno, Giovanni Simonetti

**Affiliations:** ^1^Department of Diagnostic Imaging and Interventional Radiology, Molecular Imaging and Radiotherapy, Fondazione Policlinico “Tor Vergata”, Viale Oxford 81, 00133 Rome, Italy; ^2^Department of Biopathology and Image Diagnostics, Fondazione Policlinico “Tor Vergata”, Viale Oxford 81, 00133 Rome, Italy

## Abstract

*Aim*. To compare the diagnostic performance of diffusion weighted imaging (DWI) using *b*-values of 1000 s/mm^2^ and 2000 s/mm^2^ at 3 Tesla (T) for the evaluation of clinically significant prostate cancer. *Matherials and Methods*. Seventy-eight prostate cancer patients underwent a 3T MRI scan followed by radical prostatectomy. DWI was performed using *b*-values of 0, 1000, and 2000 s/mm^2^ and qualitatively analysed by two radiologists. ADC maps were obtained at *b*-values of 1000 and 2000 s/mm^2^ and quantitatively analyzed in consensus. *Results*. For diagnosis of 78 prostate cancers the accuracy of DWI for the young reader was significantly greater at *b* = 2000 s/mm^2^ for the peripheral zone (PZ) but not for the transitional zone (TZ). For the experienced reader, DWI did not show significant differences in accuracy between *b*-values of 1000 and 2000 s/mm^2^. The quantitative analysis in the PZ and TZ was substantially superimposable between the two *b*-values, albeit with a higher accuracy with a *b*-value of 2000 s/mm^2^. *Conclusions*. With a *b*-value of 2000 s/mm^2^ at 3T both readers differentiated clinical significant cancer from benign tissue; higher *b*-values can be helpful for the less experienced readers.

## 1. Introduction

Prostatic adenocarcinoma is the most common cancer in men and the second leading cause of cancer deaths [1].

Actually many patients suffering from prostate cancer die with prostate cancer and not because of prostate cancer itself.

The standard of care is therefore to achieve an early diagnosis in patients with clinically significant prostate cancer (e.g., Gleason score ≥ 3 + 3).

Largest series concerning prostate cancer screening by use of PSA have shown no significant effect on the reduction of mortality [2, 3].

Clinically significant prostate cancer detection using transrectal ultrasound (TRUS) is not easy.

In a recent study from Spajic et al. on prostate TRUS examination in a large cohort of patients affected with prostate cancer, 60.6% of cancerous lesions were hypoechoic, 31.8% were isoechoic, and 7.6% hyperechoic, which is about 40% of TRUS prostate cancer missing detection [4].

Prostate multiparametric magnetic resonance imaging (mp-MRI) can be helpful for targeted biopsy, in order to detect, localize, and locally stage prostate cancer.

In the mp-MRI, diffusion-weighted imaging (DWI) can provide qualitative and quantitative informations about tumor cellularity and tissue structure and can be a useful tool for the detection and staging of prostate cancer in clinical practice [5].

DWI with a *b*-value of 800–1000 s/mm^2^ is currently recommended for prostate multiparametric MRI protocol by the European Society of Urogenital Radiology [6].

However, using these *b*-values, the prostate normal parenchyma sometimes shows a very high signal intensity, so that it could be difficult to distinguish it from prostate cancer foci.

This led to the use of higher  *b*-values that could provide higher accuracy, minimizing T2 weighted and perfusion effects, although with a decrease of the signal-to-noise (SNR) ratio and an increased susceptibility artifact and image distortion.

For these reasons it is not yet clear what is the optimal *b*-value for the evaluation of prostate carcinoma.

The aim of this retrospective study was to compare the results between a young and an experienced reader on diffusion-weighted images and ADC maps obtained with high *b*-values (1000 and 2000 s/mm^2^) using a 3 Tesla (T) clinical MRI system, correlating DWI imaging with the histological findings after radical prostatectomy.

## 2. Material and Methods

### 2.1. Patients

Between October 2011 and July 2013, 89 patients underwent 3 T MR imaging and were scheduled for radical prostatectomy in the following 4 months. This retrospective single-institution study was approved by our ethical committee, and written informed consent was obtained from each patient. Nine of these patients were excluded from the study because of a time interval of more than 4 months between MR imaging and surgery. Two patients with a poor-quality ADC map due to motion artifacts or biopsy-related hemorrhage were excluded because of potentially spurious ADC values. Thus, a total of 78 patients (mean age: 69 years; range: 45–81 years) were included in our study.

### 2.2. MR Imaging Technique

All the subjects were examined using a 3 T MR scanner (Intera Achieva, Philips Healthcare, Best, The Netherlands) with a 6-channel phased array pelvic coil for signal reception. All patients underwent DWI sequence as a part of the routine prostatic MR protocol used in our institution. Peristalsis was suppressed by intravenous administration of 20 mg of butylscopolamine bromide (Buscopan; Boehringer Ingelheim Pharma, Germany).

**Figure 1:**
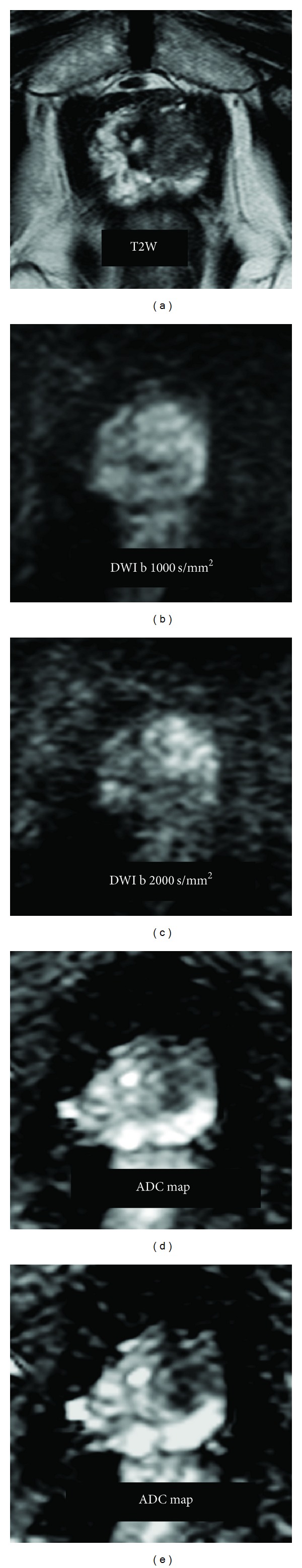
A 71-year-old man with prostate cancer Gleason 4 + 3. (a) T2-weighted image on the axial plane shows a hypointense focal area on left apex in the transitional zone. (b) DWI *b*-value 1000 s/mm^2^ shows a slight increased signal in the left transitional zone. (c) DWI *b*-value 2000 s/mm^2^; signal-to-noise ratio is decreased but signal intensity between the tumor and benign tissue is more evident. ((d) and (e)) ADC maps obtained with *b*-values of 0–1000 s/mm^2^ (d) and 0–2000 s/mm^2^ (e) show the tumor as a focal area of decreased signal intensity on the left apex in the transitional zone.

**Figure 2:**
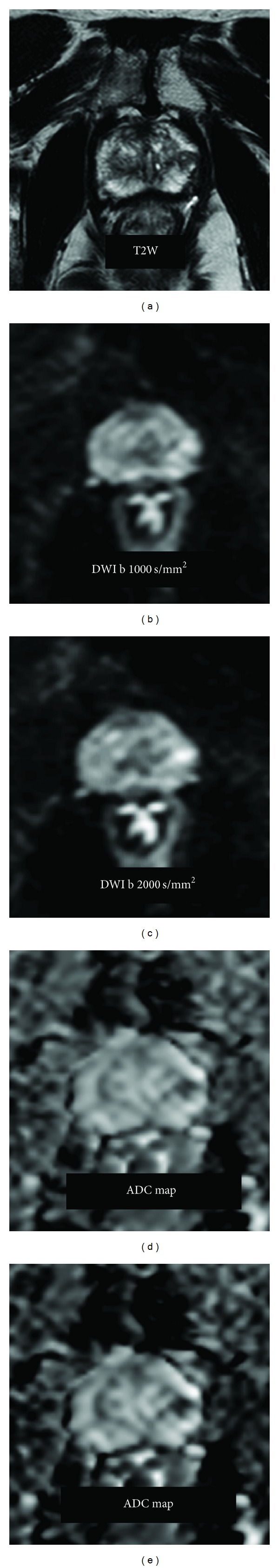
A 69-year-old man with prostate cancer Gleason 3 + 4. (a) T2-weighted image shows a hypointense focal area on left midgland in the peripheral zone. (b) DWI with *b*-value of 1000 s/mm^2^ shows a slight focal increased signal in the left peripheral zone almost indistinguishable compared to surrounding benign tissue. (c) DWI *b*-value of 2000 s/mm^2^; the tumor is easily identifiable compared to the benign tissue. ((d) and (e)) ADC maps obtained with *b*-values of 0–1000 s/mm^2^ (d) and 0–2000 s/mm^2^ (e) show the tumor as a focal area of decreased signal intensity on the left midgland in the peripheral zone.

**Figure 3:**
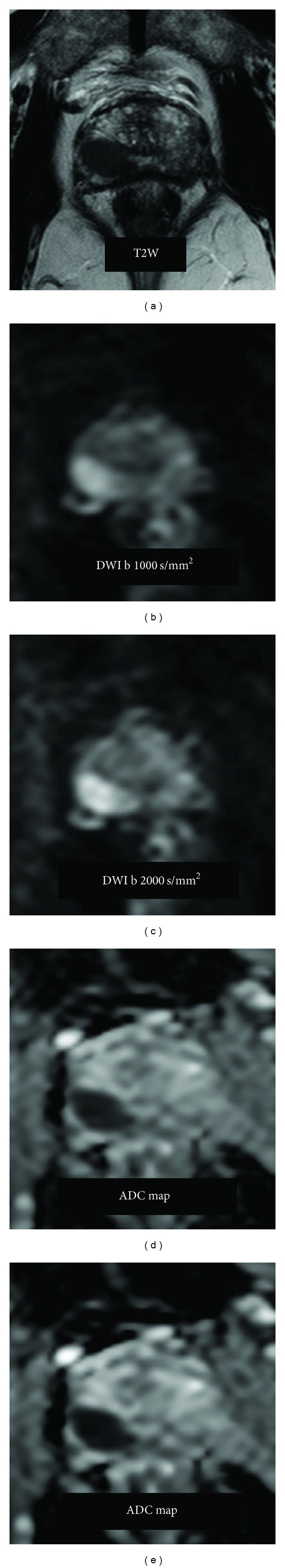
A 74-year-old man with prostate cancer Gleason 4 + 4. (a) T2-weighted image shows a hypointense focal area on right midgland in the peripheral zone. ((b) and (c)) DWI *b*-value of 1000 s/mm^2^ and *b*-value of 2000 s/mm^2^ show a focal increased signal in the right peripheral zone easily distinguishable from the surrounding benign tissue. ((d) and (e)) ADC maps obtained with *b*-values of 0–1000 s/mm^2^ (d) and 0–2000 s/mm^2^ (e) show the tumor as a focal area of decreased signal on the right midgland of the peripheral zone.

Turbo spin-echo T2-weighted images in three orthogonal planes (Figures 1(a), 2(a), and 3(a)) and T1W axial images were acquired.

Axial DWI was obtained using a modified Stejskal-Tanner spin-echo echo-planar imaging (EPI) sequence with the following parameters: TR/TE 2500/65 ms; flip angle 90; NEX 3; *b*-values 0, 1000 (Figures 1(b), 2(b), and 3(b)), and 2000 (Figures 1(c), 2(c), and 3(c)) s/mm^2^; matrix 128 × 128; FOV AP 160 mm × RL 144 mm × FH 69 mm; and slice thickness: 3/0 mm for covering the entire prostate and seminal vesicles.

Motion-probing gradients (MPGs) were applied in three orthogonal orientations for ADC calculation, with a scan time of less than 5 minutes. Both axial T2W and DWI were obtained with slice position and thickness of 3 mm. An acceleration factor of 2 was applied using the modified sensitivity encoding (mSENSE) parallel imaging technique.

ADC maps were automatically constructed on a pixel-by-pixel basis using the formula

(1) $$\begin{array}{c} \text{ADC}=\frac{\log\left[S\left(b_{1}\right)/S\left(b_{2}\right)\right]}{\left(b_{2}-b_{1}\right)}\text{,} \end{array}$$

where ADC is the molecular diffusion coefficient, *S*(*b*_1_) and *S*(*b*_2_) are the signal intensities of the diffusion weighting gradients obtained using different *b*_1_- and *b*_2_-values, and *b* is the diffusion-weighted factor expressed as seconds per square millimeter. ADC values were calculated for a pair of *b*-values: 0 and 1000 s/mm^2^ (Figures 1(d), 2(d), and 3(d)) and 0 and 2000 s/mm^2^ (Figures 1(e), 2(e), and 3(e)).

### 2.3. Histopathologic Examination

In all 78 patients, prostate cancer was proven histopathologically after radical prostatectomy. All the specimens were marked with ink and fixed overnight in 10% buffered formalin. Transverse step sections were cut at 3 to 4 mm intervals in a plane perpendicular to the prostatic urethra. The apex and base were sliced sagittally to assess the caudal and cranial surgical margins. All the slides obtained from the whole-mount pathologic step-section slices were reviewed by two experienced pathologists who were unaware of the MRI findings. The reviewer recorded the size, location, and Gleason scores (GSC) of all tumor foci on a standardized diagram of the prostate.

### 2.4. Imaging Analysis

All MR images were archived using a picture archiving and communication system (PACS; PathSpeed Workstation; GE Medical Systems, Milwaukee, WI, USA). Two radiologists, one experienced reader and one young reader, who were unaware of the clinical, surgical, and histological findings, analyzed the MR images retrospectively, the experienced reader with more than 900 mp-MR prostate examinations readings and the young reader with approximately 150 prostate mp-MRI readings at the time of the study.

The readers identified and analysed only the largest lesion on the image set acquired.

In addition both readers measured the maximal diameter of the largest lesion.

For qualitative analysis, prostate gland was divided into 24 prostate sectors: base, midgland, and apex (right, left, anterior, and posterior) in the peripheral zone (PZ) and base, midgland, and apex (right, left, anterior, and posterior) in the transition one (TZ). The blinded readers were independently asked to identify the presence or absence of cancer on DWI.

For qualitative analysis, basing on the anatomical details of T2WI, index DWI at *b* = 0, 1000, and 2000 s/mm^2^ was scored using a five-point scale: 1, definitely benign; 2, probably benign; 3, indeterminate; 4, probably cancer; and 5, definitely cancer; the results from each reader were compared. The diagnostic criteria for cancer on DWI was high focal signal on DWI compared to the benign tissue and low focal signal on ADC maps compared to the benign tissue.

For quantitative analysis the two readers in consensus draw regions of interest (ROIs) on the DWI with a *b*-value of 0 s/mm^2^ referring to both histopathologic findings and T2-weighted images. T2-weighted images were used to detect cancer. Malignant focal lesions of ≥5 mm in maximal diameter in the PZ and TZ of the histopathologic specimen whole mounted step section were included in this study, taking into account specimen thickness and spatial resolution of the DWI sequence. Nonmalignant tissue was carefully selected with three ROIs at PZ as well as TZ level in each patient. The largest possible oval ROIs were drawn on T2W sequence for malignant tumors (15–74 mm^2^) and normal tissues (>40 mm^2^) in both the PZ and TZ for each patient.

These ROIs were then automatically superimposed on ADC maps obtained with *b*-values of 0 and 1000 s/mm^2^ and 0 and 2000 s/mm^2^, respectively. The average ADC value within each ROI was then calculated.

### 2.5. Statistical Analysis

Results were expressed as mean and standard deviation (SD) for continuous variables and values and percentage for categorical variables. The unpaired Student's, *t*-test was used to assess differences in the ADC values between malignant and normal tissue in both the PZ and TZ.

Statistical analyses included calculations of sensitivity, specificity, and positive predictive value (PPV) in the localization of prostate cancer by dichotomizing the readings. Scores of 3 to 5 were considered “present.” The receiver-operating characteristic (ROC) curves analysis was performed to evaluate the accuracy of ADC to determine the optimal ADC cut-off values that would offer the best discrimination between malignant and normal tissue and allow comparison in the performance of the two data sets (*b*-values: 0 and 1000 s/mm^2^, 0 and 2000 s/mm^2^). Data were analyzed using MedCalc version 11.3.3.0 (MedCalc Software, Inc.; Mariakerke, Belgium).

## 3. Results

The 78 patients were found to have 109 malignant foci with a maximal transverse diameter of ≥5 mm in surgical specimens. A total of 100 of these 109 malignant foci (92.4%) could be detected by T2-weighted images, in the light of the results of prostatectomy. The mean maximal tumor size was 11.7 mm (range: 5–30 mm). Of these 109 malignant tumors, 70 were located in the PZ and 37 in the TZ; the remaining 2 were expressed in both the TZ and PZ. Because the greater part of these 2 tumors were located in both zones occupied by the PZ, they were defined as PZ cancer. ADC values for malignant and normal tissues in (a) the PZ and (b) the TZ with two sets of  *b*-values (0 and 1000 s/mm^2^, 0 and 2000 s/mm^2^) were reported in Table 1.

We analyzed only the largest lesions (*n* = 78) both for qualitative and quantitative assessment of DW images: 45 were located in the PZ and 33 in the TZ.

For qualitative analysis a significant higher diagnostic accuracy has been shown for the young reader only in the PZ using a *b*-value of 2000 s/mm^2^ compared to a *b*-value of 1000 s/mm^2^. Regarding the experienced reader, there was not a significant difference between the two *b*-values in both the PZ and the TZ. For quantitative analysis there was no significant differences for both the young and experienced reader between the two *b*-values.

**Figure 4:**
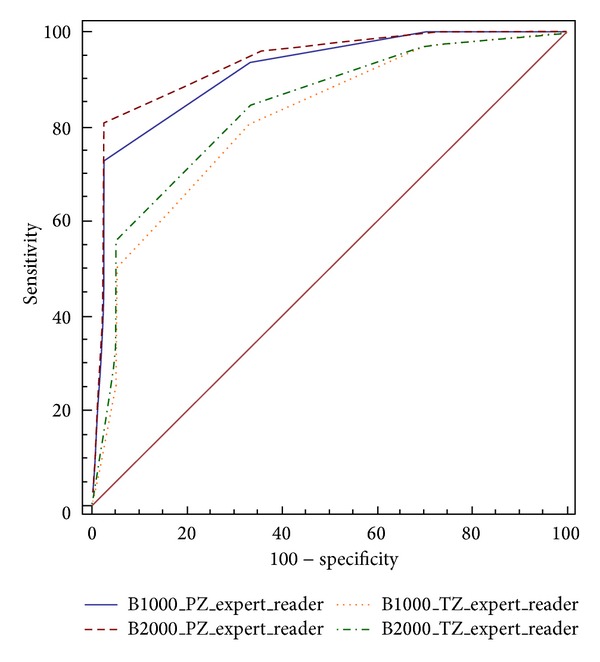
ROC curves for the experienced reader for detection of prostate cancer in the PZ (blue and red lines) and TZ (yellow and green lines) using native DWI images at *b*-values of 1000 s/mm^2^ and 2000 s/mm^2^.

For qualitative analysis, using ROC curve analysis, among PZ cancers, readings by expert reader revealed a diagnostic accuracy of 91% at DWI value with *b* = 0–1000 s/mm^2^ and a diagnostic accuracy of 94% using a DWI value with *b* = 0–2000 s/mm^2^. Differences among the two sets were not statistically significant (*P* = 0.07) (Figure 4). Moreover, among TZ cancers readings by expert reader, ROC curve analysis revealed diagnostic accuracy of 81% using a DWI value with *b* = 0–1000 s/mm^2^ and a diagnostic accuracy of 83% at DWI value with *b* = 0–2000 s/mm^2^. The differences among the two sets were not statistically significant (*P* = 0.13) (Figure 4).

**Figure 5:**
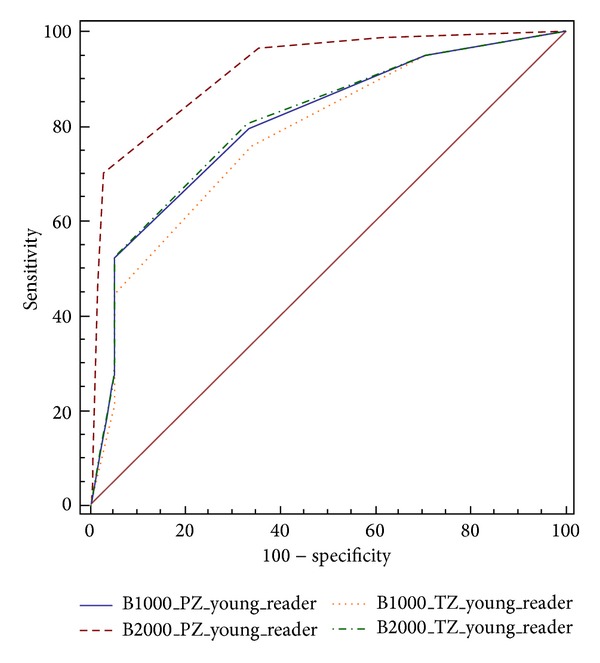
ROC curves for the young reader for detection of prostate cancer in the PZ (blue and red lines) and TZ (yellow and green lines) using native DWI images at *b*-values of 1000 s/mm^2^ and 2000 s/mm^2^.

For the young reader, among the PZ cancers, ROC curve analysis revealed for DWI value with *b* = 0–1000 s/mm^2^ a diagnostic accuracy of 79% and using a DWI value with *b* = 0–2000 s/mm^2^ a diagnostic accuracy of 93%. The differences among the two sets were statistically significant (*P* = 0.001) (Figure 5). Indeed, among TZ cancers readings by young reader, the differences among the two sets were not statistically significant (*P* = 0.16) (Figure 5). ROC curve analysis revealed at DWI value with *b* = 0–1000 s/mm^2^ diagnostic accuracy of 78% and using a DWI value with *b* = 0–2000 s/mm^2^ a diagnostic accuracy of 81%.

**Figure 6:**
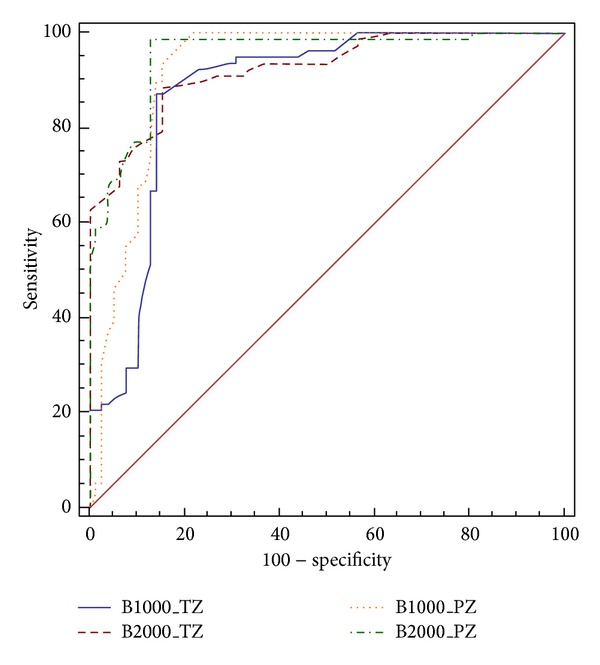
ROC curves for prostate cancer detection in the PZ (yellow and green lines) and TZ (blue and red lines) using ADC maps at *b*-values of 0–1000 s/mm^2^ and 0–2000 s/mm^2^.

For quantitative analysis, among the PZ cancers, ROC curve analysis revealed a diagnostic accuracy of 0.921 for ADC value with *b* = 0–1000 s/mm^2^, and using a cut-off value 1.43 × 10^−3^ mm^2^/s showed a sensitivity of 93.6% and a specificity of 84.6%, with a PPV of 85.9%. An ADC value with *b* = 0–2000 s/mm^2^ showed a diagnostic accuracy of 0.952, and using a cut-off value of 1.22 × 10^−3^ mm^2^/s showed a sensitivity of 98.7% and a specificity of 87.2% with a PPV of 88.5%. The differences among the two sets were not statistically significant (*P* = 0.12) (Figure 6).

Among TZ cancers, ROC curve analysis revealed that an ADC value with *b* = 0–1000 s/mm^2^ had a diagnostic accuracy of 0.878 and using a cut-off value of 1.18 × 10^−3^ mm^2^/s showed a sensitivity of 87.2% and a specificity of 85.9% with a PPV of 86.1%. ADC values with *b* = 0–2000 s/mm^2^ showed a diagnostic accuracy of 0.925. At a cut-off value of 0.88 × 10^−3 ^mm^2^/s it showed a sensitivity of 88.46% and a specificity of 84.62% with a PPV of 85.2%. The differences among the two sets were not statistically significant (*P* = 0.06) (Figure 6).

## 4. Discussion

The aim of our study was to standardize DW-MRI protocol, as regards the *b*-value, for the qualitative and quantitative evaluation of prostate cancer in common clinical practice without contrast agent administration.

It is widely debated in literature which could be the best *b*-value for prostate cancer detection in order to highlight the tumor tissue, reducing the signal from benign prostate tissue, in order to obtain good quality ADC maps for better measurements and visual imaging interpretations [6] without increasing the acquisition time or reduce the signal-to-noise ratio. DWI sequences are included in the standard mp-MRI protocol of the prostate for both detection and local staging [7–9].

However, in literature, there are large differences in both the analysis of DW images (evaluation of native DW images, ADC maps, or both; expert or young readers) and the results.

Kim et al. [10] and Koo et al. [11] in their research reported a *b*-value of 1000 s/mm^2^ showing higher sensitivity of the ADC maps obtained at a *b*-value of 1000 s/mm^2^ than those obtained with a *b*-value of 2000 s/mm^2^. Regarding the specificity, Kim et al. [10] stated no significant difference between the two *b*-values. Koo et al. [11] demonstrated a higher specificity of the ADC maps obtained with a *b*-value of 2000 s/mm^2^ than those obtained at a *b*-value of 1000 s/mm^2^.

On the contrary, other papers [12–18] reported a *b*-value of 2000 s/mm^2^ as recommendable in prostate cancer detection, but in these articles there is some inhomogeneity regarding the analysis of images and results.

Katahira et al., Rosenkrantz et al., Ohgiya et al., and Ueno et al., [12–15], analysing native DW images, showed that as preferable the use of a *b*-value of 2000 s/mm^2^ compared to a *b*-value of 1000 s/mm^2^. Metens et al. [16] underlines that native DW images with a *b*-value of 2000 s/mm^2^ have better contrast-to-noise ratio (CNR) in comparison with a *b* value of 1000 s/mm^2^ but lower than those with a *b*-value of 1500 s/mm^2^.

Rosenkrantz et al. [13], in the evaluation of ADC maps, emphasize the two *b*-values (1000 s/mm^2^ and 2000 s/mm^2^) as substantially superimposable, whereas Kitajima et al. [17] showed in the peripheral zone ADC maps at *b*-value 2000 s/mm^2^ little diagnostic advantage in comparison with a *b*-value of 1000 s/mm^2^, although more recently he reported a significant advantage [18].

In the studies cited above the experience of readers who analyzed images was different, and in only two cases [11, 12] image analysis was performed by young readers, one of which evaluated only native DW images [12] and the other only ADC maps [11].

In our study, as in the study published by Rosenkrantz et al. [13], we evaluated both native DW images and ADC maps, analyzed by both a young reader and an experienced reader, to assess the utility of using higher *b*-value for less experienced readers.

The qualitative evaluation for the young reader showed a significantly greater accuracy in DWI of peripheral zone (PZ) with *b* value 2000 s/mm^2^ compared to 1000 s/mm^2^.

In the transitional zone (TZ) we did not find a significant difference between the two *b* values analyzed, although it was higher for a *b*-value of 2000 s/mm^2^.

For the experienced reader there was not a significant difference between PZ and transition zone, although a greater utility of 2000 s/mm^2^ in the PZ was reported.

Regarding qualitative analysis of DW images, the use of higher *b*-values is useful for less experienced radiologists; best signal suppression of benign prostate tissue and greater evidence of signal restriction with a higher *b*-value allow an immediate diagnostic evaluation of the images. Images with a *b*-value of 1000 s/mm^2^ cannot suppress benign tissue in the PZ and sometimes obscure tumor lesions due to persistent T2-shine-through effects [19]. This aspect needs to be elicitated as the great spread of prostate cancers requires an increase in MRI examinations for diagnosis, local staging, lesions targeting for biopsy, or focal therapies, so that the interpretation of the mp-MRI must be easy in clinical practice, without the need of great experience.

As for quantitative evaluation with ADC maps, a higher diagnostic accuracy was obtained with a *b*-value of 2000 s/mm^2^ compared to 1000 s/mm^2^, although it was not statistically significant. The value of ADC for both benign and pathological tissues decreases when the *b*-value used increases [16]. ADC measurements cannot differentiate low-grade tumors from benign tissue [20], but that is not a problem because mp-MRI of the prostate aims to detect clinically significant tumors. It is therefore important to emphasize that our study, in agreement with other studies, shows that ADC value in both PZ and transitional zone (TZ) is significantly lower in intermediate or high grade tumours (Gleason ≥ 3 + 3) compared to benign tissue [16, 21].

As a limitation, this study was retrospective; further prospective studies are therefore needed. We did not use the endorectal coil which could increase the signal-to-noise ratio of the PZ, in order to reduce examination time, patients discomfort, and probe artifacts. We did not calculate the signal-to-noise ratio of DWI images with different *b*-values. Finally, a correlation of MRI with histological findings was not always easy because of movement artifacts on diffusion weighted sequences.

In conclusion we found a high accuracy of DWI as regards both the quantitative and qualitative analysis.

DWI sequences with a *b*-value of 2000 s/mm^2^ are more accurate than those with a *b*-value of 1000 s/mm^2^ in assessing tumor lesions from prostate cancer in particular for the qualitative evaluation and significantly in the PZ for young readers.

The ADC maps obtained with a value of 2000 s/mm^2^ are more accurate than those obtained with a *b*-value of 1000 s/mm^2^, although without statistically significant differences.

For both the qualitative and quantitative evaluation, the diagnostic accuracy of DWI in PZ is higher than in TZ.

The use of high *b* value can be of great help especially for less experienced readers.

## Figures and Tables

**Table 1:** The ADC values of malignant and benign peripheral and transitional tissue at *b* = 0–1.000 and 0–2.000 s/mm^2^.

ADC values (×10^−3^ mm^2^/s)	Malignant	Benign	*P* value
PZ tissue			
*b* = 0.1000 s/mm^2^			
Mean (SD)	1.15 ± 0.2	1.61 ± 0.3	<0.001
Range	0.7–1.45	0.7–2.3	
*b* = 0.2000 s/mm^2^			
Mean (SD)	0.80 ± 0.25	1.37 ± 0.19	<0.001
Range	0.47–1.5	0.75–1.67	

TZ tissue			
*b* = 0.1000 s/mm^2^			
Mean (SD)	0.98 ± 0.2	1.35 ± 0.23	<0.001
Range	0.7–1.4	0.8–1.8	
*b* = 0.2000 s/mm^2^			
Mean (SD)	0.70 ± 0.16	1.09 ± 0.22	<0.001
Range	0.45–1.08	0.75–1.48	
